# Assimilation of Web-Based Urgent Stroke Evaluation: A Qualitative Study of Two Networks

**DOI:** 10.2196/medinform.3028

**Published:** 2014-04-15

**Authors:** Rajendra Singh, Lars Mathiassen, Jeffrey A Switzer, Robert J Adams

**Affiliations:** ^1^Arnold School of Public HealthHealth Services Policy and ManagementUniversity of South CarolinaColumbia, SCUnited States; ^2^Robinson College of BusinessCenter for Process InnovationGeorgia State UniversityAtlanta, GAUnited States; ^3^Medical College of GeorgiaDepartment of NeurologyGeorgia Regents UniversityAugusta, GAUnited States; ^4^Medical University of South CarolinaDepartment of NeuroscienceMedical University of South CarolinaCharleston, SCUnited States

**Keywords:** telemedicine, stroke, telestroke, information technology assimilation, case study

## Abstract

**Background:**

Stroke is a leading cause of death and serious, long-term disability across the world. Urgent stroke care treatment is time-sensitive and requires a stroke-trained neurologist for clinical diagnosis. Rural areas, where neurologists and stroke specialists are lacking, have a high incidence of stroke-related death and disability. By virtually connecting emergency department physicians in rural hospitals to regional medical centers for consultations, specialized Web-based stroke evaluation systems (telestroke) have helped address the challenge of urgent stroke care in underserved communities. However, many rural hospitals that have deployed telestroke have not fully assimilated this technology.

**Objective:**

The objective of this study was to explore potential sources of variations in the utilization of a Web-based telestroke system for urgent stroke evaluation and propose a telestroke assimilation model to improve stroke care performance.

**Methods:**

An exploratory, qualitative case study of two telestroke networks, each comprising an academic stroke center (hub) and connected rural hospitals (spokes), was conducted. Data were collected from 50 semistructured interviews with 40 stakeholders, telestroke usage logs from 32 spokes, site visits, published papers, and reports.

**Results:**

The two networks used identical technology (called Remote Evaluation of Acute isCHemic stroke, REACH) and were of similar size and complexity, but showed large variations in telestroke assimilation across spokes. Several observed hub- and spoke-related characteristics can explain these variations. The hub-related characteristics included telestroke institutionalization into stroke care, resources for the telestroke program, ongoing support for stroke readiness of spokes, telestroke performance monitoring, and continuous telestroke process improvement. The spoke-related characteristics included managerial telestroke championship, stroke center certification, dedicated telestroke coordinator, stroke committee of key stakeholders, local neurological expertise, and continuous telestroke process improvement.

**Conclusions:**

Rural hospitals can improve their stroke readiness with use of telestroke systems. However, they need to integrate the technology into their stroke delivery processes. A telestroke assimilation model may improve stroke care performance.

## Introduction

Stroke is a leading cause of death and serious, long-term disability in the United States. In 2008, nearly 800,000 people suffered a stroke, resulting in the deaths of more than 134,000 people [1]. Stroke-related costs are also very high—in 2007, the estimated mean lifetime costs resulting from stroke in the United States were $140,000 per patient and the estimated total costs were $62.7 billion [2]. Worldwide, 15 million people suffer stroke each year; of these, 5 million die and another 5 million are permanently disabled [3].

For ischemic (ie, nonbleeding) strokes, a blood-clot dissolving drug tissue plasminogen activator (tPA) greatly reduces the risk of severe disabilities if administered within 4 ½ hours from the onset of stroke symptoms [4,5]. However, for nonischemic (ie, hemorrhagic) strokes, the tPA treatment would be fatal to the patient. The clinical diagnosis of stroke is therefore challenging; emergency physicians may have difficulty differentiating an ischemic stroke from conditions with a similar presentation and determining which patients would benefit from tPA. Therefore, urgent stroke diagnosis requires readily available neurological expertise, which puts rural hospitals in the difficult position of either transferring all stroke patients to regional medical centers or acquiring such expertise at the risk of variable demand and negative budget impacts.

Information technology (IT)—in the form of specialized Web-based telemedicine systems that include videoconferencing and supporting applications that enable a remote stroke specialist to view and evaluate a patient—has helped address the challenge of urgent stroke care in underserved communities [6]. Such systems, referred to as telestroke, allow emergency departments (EDs) in hospitals to receive patients with suspected stroke and to quickly determine (after consulting a remote stroke specialist) whether to administer tPA [7,8]. Consequently, rural hospitals can offer patients the same emergency stroke care as larger hospitals, provided they collaborate with the larger hospital through telestroke. Despite these technological advancements, telestroke systems in rural hospitals remain underutilized. This may explain, in part, why systemic treatment of stroke patients with tPA remains very low—reportedly between 3% and 5% nationally [9]. This research examines the postdeployment utilization of telestroke across EDs of participating rural hospitals in 2 telestroke networks. In particular, this research explains variations in utilization of a Web-based telestroke system for urgent stroke evaluation.

IT utilization (or assimilation) can be defined as “the extent to which the use of technology diffuses across the organizational projects or work processes and becomes routinized in the activities of those projects and processes” [10]. Following Cooper and Zmud’s [11] six-stage model of IT implementation process, IT assimilation combines routinization (when IT application usage is encouraged as a normal activity) and infusion (when increased organizational effectiveness results from using the IT application to its fullest potential). Before IT assimilation can occur, the organization must already have completed the earlier stages of IT implementation. These stages include initiation (when the organization has scanned its problems, opportunities, and available IT solutions, and found a match between an IT solution and its application), adoption (when the organization has decided to invest resources to implement the IT solution), adaptation (when the IT application has been developed, installed, and made available for use), and acceptance (when organizational members have committed to using the IT application) [11]. Thus, IT assimilation occurs when an organization progresses beyond initial technology deployment and integrates it into day-to-day work processes to enhance business performance [12-14].

Recent studies have explored IT adoption in health care organizations [15-19], but Fichman and Kemerer [20], Zhu et al [13], and others have noted that adoption does not always result in effective assimilation of the technology. Still, relatively few studies have explored IT assimilation in health care organizations. Notable examples include Meyer and Goes’ [21] nine-stage model of assimilation of technological innovations in hospitals, Ash’s [22] investigation of assimilation (“internal diffusion and infusion”) of three technological innovations across 67 academic health science centers, Chau and Hu’s [23] study of telemedicine assimilation in hospitals, Leonard and Sittig’s [24] IMPROVE-IT model connecting IT utilization to health outcomes, and Davidson and Heslinga’s [25] examination of assimilation of electronic health records in physician practices. Despite these and a few other IT assimilation studies in health care organizations, there are no in-depth examinations of variations in assimilation of a particular technology across hospitals.

Recent telestroke literature has focused on the organizational, managerial, financial, technical, and legal issues that influence adoption. The enablers of telestroke adoption include a stroke systems of care model with primary and comprehensive stroke centers of excellence, statewide and local stroke champions, pre-hospital and in-hospital coordination, favorable regulatory and reimbursement policies, stakeholder support and communication, and appropriate IT infrastructure [26-30]. The barriers to telestroke adoption include lack of public awareness of stroke symptoms and the need for timely treatment, logistical and coordinative challenges of providing appropriate and timely treatment, limited availability of local neurologists, physician reluctance to use tPA, regulatory and jurisdictional issues, technical and financial issues, and lack of stakeholder support [29-32]. However, to our knowledge, no studies have explored factors that enable telestroke assimilation (ie, postdeployment utilization) in hospitals. Hence, the aim of this study was to examine potential sources of variations in telestroke assimilation in hospitals that offer urgent stroke evaluation and management in collaboration with a tertiary hospital.

## Methods

### Research Design and Case Context

Based on purposive sampling [33], we organized this research as an exploratory, qualitative case study of 2 stroke networks in Georgia and South Carolina. Each network includes a hub—a comprehensive stroke center at the Georgia Regents University (GRU) and at the Medical University of South Carolina (MUSC)—and connected spokes (ie, rural hospitals supported by the hub). The two networks use the same technology (Remote Evaluation of Acute isCHemic stroke, REACH), they are of similar size and complexity (17 and 15 spokes, respectively), and they operate in similar contexts (providing services to EDs in rural hospitals in the southeast United States). This design allowed us to conduct cross-case comparisons [33,34] of how hub-related characteristics may influence telestroke assimilation across spokes.

**Figure 1:**
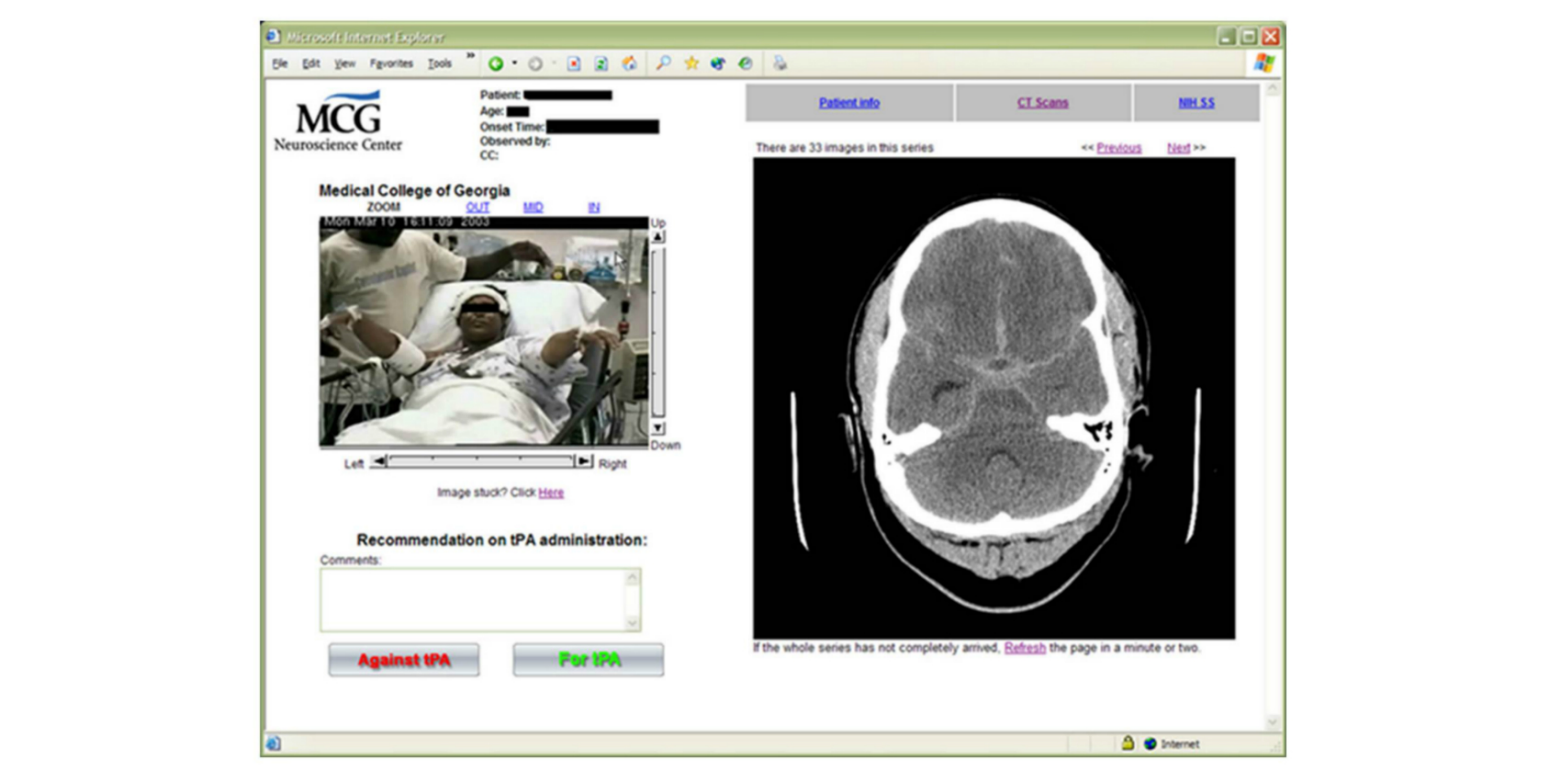
REACH Web interface showing a patient's CT scan.

Recognizing the potential of using telestroke to link hub-based specialists to rural hospitals, a team of GRU neurologists developed the REACH system. The system comprised a mobile, Internet-ready REACH cart (with a mounted adjustable camera, a phone, and a high-resolution monitor) that could be wheeled into the ED room where the stroke patient was being examined. As shown in Figure 1, the software embedded within the cart included a Web-based interface to view and share computed tomography (CT) scans and other patient-related information stored within the hospital’s electronic medical record system (EMR), picture archiving and communication system, and laboratory information system. In February 2003, GRU signed a contract with the first spoke where it placed a REACH cart. The spoke ED staff activated the REACH system if a patient with suspected stroke arrived within 4 hours of onset of symptoms and then contacted the on-call stroke specialist. The specialist logged onto REACH website via any broadband Internet-connected computer and completed the consultation with a recommendation to administer (or not to administer) tPA to the patient. A for-profit company (REACH Health Inc) provided round-the-clock technology support. By August 2012, 17 hospitals had joined the GRU-REACH network. The MUSC-REACH network was established when one of the founders of REACH joined MUSC and set up a telestroke program in South Carolina in May 2008. By August 2012, 15 hospitals had joined MUSC-REACH. The design, technical details, outcomes, and organizational challenges of REACH have been published elsewhere [26,35-47].

### Data Sources

We collected primary data between March and August 2012 by visiting the 2 hubs and 8 selected spokes (Table 1). These spokes—4 in each network—were selected (out of 32) based on REACH utilization; they included spokes with higher than average and lower than average REACH utilization in the network. During our field visits, we interviewed key stakeholders associated with telestroke, such as administrators, managers, ED physicians, nurses, neurologists, and emergency medical service (EMS) representatives. We asked all respondents to share their experiences of using REACH. The semistructured interviews lasted about 1 hour each. Altogether, we conducted 50 in-person and telephone interviews with 40 stakeholders. To enhance data quality, we collected evidence from multiple sources, including published papers related to the REACH network, as well as internal presentations, emails, and reports. This secondary data helped to gain insight into the current and historical context of REACH implementation in the two networks, and to validate the information collected during the interviews.

We also collected archival data from the 2 hubs related to REACH consultations with each spoke since the start of the telestroke program. To account for variations in spoke ED volume across hospitals, we adjusted the annual rate of REACH consultations at each hospital by its reported ED volume. We refer to the average adjusted annual telestroke consultation rate (calculated as number of REACH consultations/year per 10^4^ ED volume) as REACH assimilation. Thus, we consider the REACH-enabled consultation rate as a proxy for telestroke assimilation. It must be emphasized that this paper focuses on the decision-making enabled by the telestroke technology; therefore, we have examined REACH consultations rather than the resulting tPA usage.

**Table 1:** Primary and secondary data sources.

Primary data sources	Secondary data sources
15 semistructured interviews at 2 hubs (with neurologists, stroke coordinators, ED nurse managers, stroke service line manager, and data analyst)	14 published papers [26,35-47] 10 internal documents related to 2 hubs (including internal presentations, emails, reports, and meeting notes) Archival data related to REACH consultations with each spoke
30 semistructured interviews at 8 spokes (with chief executive officers and chief operations officers, stroke coordinators, neurologists, ED directors, ED physicians, ED nurses, quality managers, radiology nurses, and EMS directors) One staff meeting at a spoke	15 internal documents related to 8 spokes (including presentations, stroke protocols, emails, and meeting notes)
5 semistructured interviews at REACH Health Inc (with chief executive officer, chief technology officer, marketing director, business manager, and IT specialist) One REACH system demonstration	5 internal documents (including presentations, technical specifications, and meeting notes)

##  Results

### Network-Level Variation in Telestroke Assimilation

Table 2 shows basic information about the spokes. The 17 spokes in GRU-REACH network have 1831 beds (range 10-236, mean 108, SD 76) and receive more than 300,000 ED patients/year. Between February 2003 (when GRU-1 became a spoke) and August 2012 (when we collected the data), these spokes reported 2179 REACH consultations (range 48-280, mean 128, SD 71). The 15 spokes in MUSC-REACH network have 2482 beds (range 25-453, mean 165, SD 122) and receive more than 450,000 ED patients/year. Between May 2008 and August 2012, these spokes reported 2753 REACH-enabled consultations (range 60-411, mean 183, SD 107).

**Figure 2:**
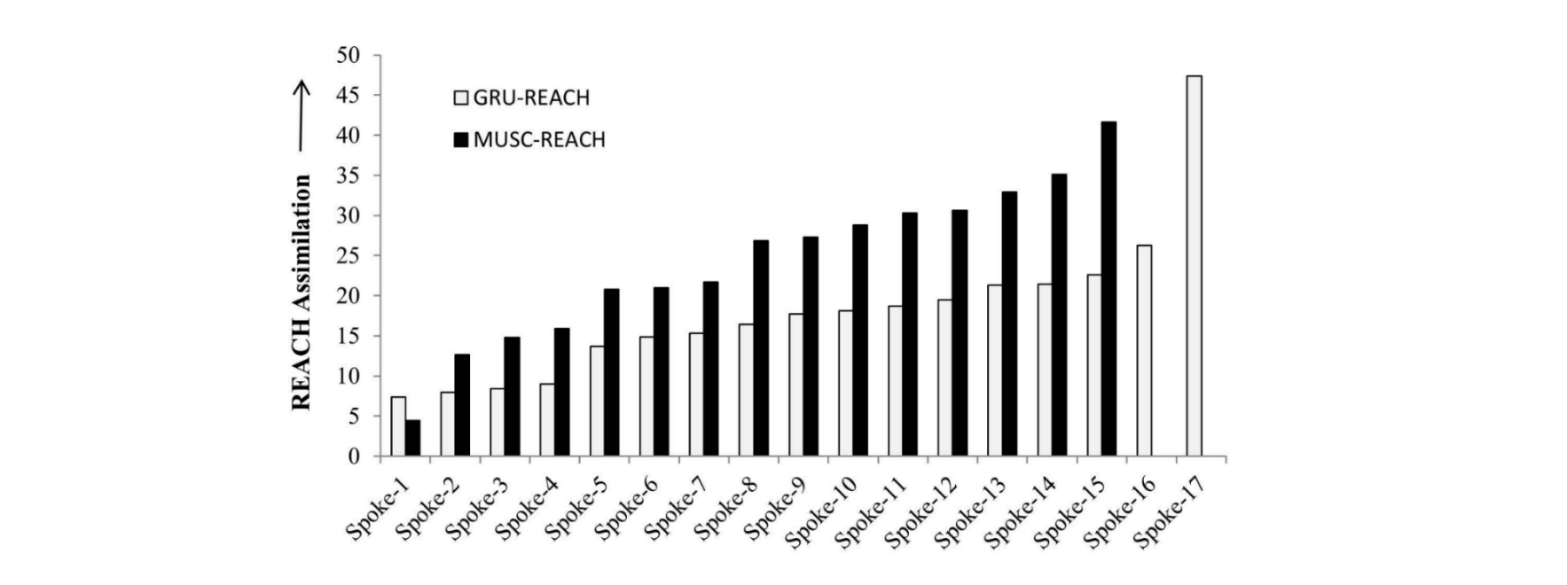
Variation in telestroke assimilation across networks.

Figure 2 compares the REACH assimilation across spokes in the two networks. Except for 1 spoke (MUSC-4 in Table 2 rarely used telestroke and left the network in November 2010 after hiring a neurologist), the MUSC-REACH network outperformed GRU-REACH with a 35% higher REACH assimilation (24.32 vs 18.01; *P*=.07). One reason is that when one of REACH’s founding neurologists joined MUSC, he leveraged the lessons learned during the development of the GRU network. This neurologist explained: “When I started the MUSC telestroke program, I did not want to make the same mistakes we did when we developed the Georgia REACH program.”

**Table 2:** Network characteristics and REACH assimilation data.

Telestroke network	Spoke hospital	Joining date	No. of beds	Primary stroke center	Stroke coordinator	Local neurologist	REACH assimilation^a^
GRU	1	2/1/03	72	No	No	No	18.70
GRU	2	3/1/03	47	No	No	No	15.31
GRU	3	7/1/03	50	No	No	No	26.24
GRU	4	8/1/03	10	No	No	No	18.12
GRU^b^	5	9/1/03	56	No	No	No	21.33
GRU	6	3/1/04	65	No	No	No	16.44
GRU	7	4/1/04	20	No	No	No	9.01
GRU^b^	8	2/1/05	52	No	No	No	17.71
GRU	9	3/1/06	71	No	No	No	13.72
GRU	10	1/1/08	191	No	No	Yes	7.93
GRU	11	8/1/08	236	Yes	No	Yes	7.35
GRU	12	6/1/09	40	No	No	No	19.51
GRU	13	10/1/09	190	Yes	Yes	Yes	21.42
GRU^b^	14	10/1/09	196	Yes	Yes	Yes	22.65
GRU	15	1/1/10	180	Yes	Yes	Yes	47.40
GRU	16	3/1/10	163	No	No	Yes	8.39
GRU^b^	17	11/1/10	192	No	Yes	Yes	14.88
MUSC	1	5/1/08	131	No	No	Yes	20.81
MUSC	2	5/6/08	140	No	No	Yes	30.66
MUSC^b^	3	5/7/08	453	No	Yes	Yes	14.77
MUSC	4	9/1/08	220	No	No	No	4.47
MUSC	5	9/18/08	124	No	No	No	15.89
MUSC	6	12/23/08	25	No	Yes	No	35.10
MUSC^b^	7	1/20/10	45	Yes	Yes	Yes	41.62
MUSC^b^	8	3/26/10	288	Yes	Yes	Yes	26.84
MUSC^b^	9	5/19/10	121	No	Yes	Yes	32.93
MUSC	10	7/29/10	79	No	No	No	28.80
MUSC	11	8/26/10	231	No	No	No	21.69
MUSC	12	01/21/11	116	No	No	Yes	12.64
MUSC	13	2/28/11	105	No	No	Yes	30.30
MUSC	14	2/28/11	50	No	No	Yes	21.00
MUSC	15	3/2/11	354	No	Yes	Yes	27.30

^a^REACH assimilation calculated as number of telestroke consultations/year per 10^4^ ED volume.

^b^Spokes selected for detailed examination (through field visits).

### Hub-Level Variation in Telestroke Assimilation

Based on primary and secondary data analysis, we identified several hub-related practices that can explain the superior telestroke assimilation in the MUSC-REACH network. Table 3 presents these findings. These practices include telestroke institutionalization into stroke care, providing resources for telestroke program, support for stroke readiness of spokes, telestroke performance monitoring, and continuous telestroke process improvement.

**Table 3:** Comparison of hub-level practices.

GRU-REACH hub	MUSC-REACH hub
GRU-REACH hub invited most of the early spokes to become part of the network and subsidized their participation; most recent spokes sought membership without subsidies.	MUSC-REACH hub invited most of the early spokes to become part of the network, but participation was not subsidized; most recent spokes also sought membership without subsidies.
GRU administration considers telestroke as an ongoing experiment rooted in the vision and goodwill of the stroke specialists who developed REACH. As such, the specialists feel REACH is “taken for granted.” GRU administration does not provide support for telestroke operations.	MUSC administration considers telestroke an integral part of their neuroscience service line, and therefore provides ongoing support (including director’s pay, advertising budget, and administrative salary support for credentialing, billing, operations, and project management).
There is broad consensus among the hub stroke specialists that network performance would benefit from a full-time telestroke coordinator.	A dedicated telestroke coordinator at the hub has been part of the network from the start. She facilitates coordination and training of the spokes’ ED staff.
The hub has no established processes for reinforcing telestroke use and related routines at the spokes. There are no continuous quality improvement processes in place. Any problems related to stroke consultations are reported to REACH Health Inc with variable follow-up.	The hub has established processes for reinforcing telestroke use and related routines at the spokes. It has a formal continuous quality improvement process in place. Any problem during telestroke consultation is reported to REACH Health Inc and its resolution is coordinated by the hub staff.
The hub collects spokes’ telestroke use data, but there is no systematic analysis of the data.	The hub telestroke coordinator collects spokes’ usage data and conducts systematic analysis.
A hub stroke specialist visits spokes when they go live with REACH and at rare occasions for major upgrades. However, there are no ongoing training and follow-up procedures.	A hub telestroke specialist visits spokes when they go live with REACH and maintains regular communication (with some visits) to spokes to understand concerns and train ED staff.
The hub stroke specialists rarely conduct ongoing training for spokes.	The hub facilitates occasional breakfast meetings, lunch-and-learn, mock-consults, and dinners with spoke ED physicians and nurses to discuss issues.
The hub has no formal system to provide site-specific feedback.	The hub provides site-specific performance data. As an MUSC-REACH stroke specialist told us, “The sites love to receive such feedback.”

### Spoke-Level Variation in Telestroke Assimilation

#### Identifying Characteristics that Explain Spoke-Level Variation

Spoke-level REACH assimilation varied from 7.35 in GRU-11 to 47.4 in GRU-15 (average 18.00), and from 4.47 in MUSC-4 to 41.62 in MUSC-7 (average 24.32). We cannot explain these large variations by length of relationship with the hub or size of the spoke. For example, GRU-3 and GRU-4 joined the network within 1 month of each other, but still showed variation in assimilation (26.24 and 18.12). Moreover, GRU-13 joined the network more than 6 years after GRU-5 and both showed similar REACH assimilation (21.42 and 21.33). Furthermore, MUSC-5 and MUSC-9 had similar number of beds (124 and 121), but showed considerable variation in REACH assimilation (15.89 and 32.93).

To explain the observed variations across all spokes, we first considered the availability of local neurological expertise for post-tPA patient supervision. Seven spokes in GRU-REACH and 10 spokes in MUSC-REACH had an on-call local neurologist. As Table 4 shows, when local neurology support was available, GRU-REACH spokes showed similar assimilation (18.57 vs 17.61, *P*=.87), whereas MUSC-REACH spokes showed relatively higher assimilation (25.89 vs 21.19, *P*=.46). Overall, availability of local neurological expertise was associated with a 21.70% improvement in assimilation (22.88 vs 18.80, *P*=.24). Although these variations do not show statistical significance (the very small sample sizes may explain the *P* values generated), the data suggest that ED staff sought more telestroke consultations when a neurologist was readily available.

Next, we considered whether stroke center certification had an impact on telestroke assimilation. The US Joint Commission certifies acute care hospitals as “Primary Stroke Centers” if they have specialized knowledge and infrastructure to treat stroke patients. The certification signifies that a hospital has necessary stroke-related facilities (such as ED, EMS, and stroke unit), services (such as neurological, neuro-imaging, laboratory, and clinical support), personnel (such as acute stroke teams), practices (such as written care protocols, outcome and quality improvement activities, and continuing medical education), and commitment and support of the medical organization [48]. Overall, 4 spokes in GRU-REACH and 2 spokes in MUSC-REACH had stroke certification. As Table 4 shows, REACH assimilation was higher in these cases (54.86% higher in GRU-REACH, *P*=.38; 50.13% higher in MUSC-REACH, *P*=.38; and 43.93% higher overall, *P*=.22). Thus, the data suggest that stroke care certification resulted in higher assimilation (again, the very small sample sizes may explain the *P* values generated).

We also considered the impact of a telestroke coordinator. Such a position may help spokes establish standard processes for stroke care; collect, analyze, and use performance data to continually improve care delivery; and, become a stroke champion in the hospital and in the local community. Four spokes in GRU-REACH and 6 spokes in MUSC-REACH had a dedicated telestroke coordinator. As Table 4 shows, REACH assimilation in the spokes with stroke coordinator was significantly higher than without the coordinator (73.00% higher in GRU-REACH, *P*=.22; 43.84% higher in MUSC-REACH, *P*=.08; and 62.34% higher overall, *P*=.01), suggesting that having a dedicated coordinator resulted in higher assimilation.

To confirm and elaborate these explanations, we conducted an in-depth analysis of telestroke use at 4 selected spokes in each network. Helped by long-standing relationships with the 2 hubs, we visited these spokes and interviewed key stakeholders associated with stroke operations. These interviews provided additional insights into the current and historical context of REACH implementation at these spokes. Accordingly, we identified several notable practices that can further explain variations in telestroke assimilation across spokes.

**Table 4:** Impact of spoke characteristics on telestroke assimilation.

Spoke characteristic		REACH assimilation^a^ in GRU-REACH	REACH assimilation in MUSC-REACH	Overall REACH assimilation
**Local neurological expertise**				
	No local neurologist	17.61	21.19	18.80
	Local neurologist	18.57	25.89	22.88
	Difference (%)	5.45	22.18	21.70
**Stroke center certification**				
	No stroke certification	15.95	22.80	19.37
	Stroke certification	24.70	34.23	27.88
	Difference (%)	54.86	50.13	43.93
**Dedicated stroke coordinator**				
	No stroke coordinator	15.37	20.69	17.55
	Stroke coordinator	26.59	29.76	28.49
	Difference (%)	73.00	43.84	62.34

^a^REACH assimilation calculated as number of telestroke consultations/year per 10^4^ ED volume.

#### Local Neurological Expertise

In 6 of the 8 spokes that we visited, a combination of local neurological expertise and telestroke provided urgent stroke care. The local neurologists would follow up on patients admitted locally, including post-tPA stroke patients, in the intensive care unit (ICU). In some cases (eg, GRU-5 and MUSC-9), all emergency consultations were handled via telestroke. In other cases, local neurologists also provided acute stroke coverage in the ED either during daytime (GRU-14) or 15 days/month (GRU-17). Overall, the combination of local neurology support and REACH coverage afforded spokes expanded stroke care capability.

#### Stroke Center Certification

Three of the 8 spokes that we visited (GRU-14, MUSC-7, and MUSC-8) had received primary stroke center certification. This suggests that they had established the necessary infrastructure, acquired stroke-related specialized knowledge, and developed standardized protocols and best practices to manage urgent stroke patients. In 2004, GRU-14 became the first spoke in Georgia to receive certification. To achieve that, GRU-14 set up a dedicated stroke unit, hired three neurologists, and developed standardized protocols (such as a written “stroke code”). Prior to the stroke certification, the local EMS “dreaded bringing stroke patients to the hospital because they were not sure that the hospital had capability to deliver urgent stroke care,” and instead took the patients directly to the nearest tertiary medical center. However, as GRU-14 advertised its stroke care capabilities, the local EMS started to bring stroke patients to the hospital. Similarly, after MUSC-7 gained certification in 2010, its acceptance as the preferred stroke care center in the region increased, resulting in a growing number of stroke patients admitted at the hospital. When needed, the ED staff at these hospitals connected to GRU-hub via REACH for consultations.

#### Dedicated Telestroke Coordinator

At 6 of the 8 spokes that we visited (GRU-14, GRU-17, MUSC-3, MUSC-7, MUSC-8, and MUSC-9), a telestroke coordinator set up and developed requisite processes, and facilitated collaboration within the hospital and with the hub. The coordinator provided ongoing feedback and training to ED nurses to reinforce and improve stroke-related processes, and conducted systematic spoke performance analysis. The coordinator helped to develop best practices (such as taking blood samples for laboratory analysis while the patient was in the CT scan room), which helped to reduce delays in stroke treatment. A full-time coordinator at GRU-17 reviewed each stroke case and reported any deficiencies (eg, missed stroke diagnosis, or delays in CT scan). Spokes (eg, GRU-8) that had no dedicated telestroke coordinator used the services of a part-time coordinator. At GRU-5, MUSC-7, MUSC-8, and MUSC-9, the coordinator conducted community awareness initiatives (including health fairs, and advertisements in the local newspapers, radio, and television) to provide information about stroke symptoms and related services available at the hospital.

#### Managerial Telestroke Championship

Senior leadership support was critical to establishing and fostering telestroke capability at the spokes. In 5 of the 8 spokes that we visited (GRU-14, GRU-17, MUSC-7, MUSC-8, and MUSC-9), the senior leadership realized the value of telestroke and encouraged the ED and other staff to make it an integral part of urgent stroke care. They also provided requisite IT infrastructure and resources, and facilitated a culture of continuous improvement. In contrast, at GRU-8, several years of managerial neglect had led to a situation where the ED staff routinely referred stroke patients to other hospitals. Over time, they lost their stroke-handling skills. A nurse manager elaborated on the situation:

A few years ago, the ED staff knew what to do in case of a stroke patient. Now, I am not sure they do. I guess they don’t know when to trigger the REACH system.

#### Stroke Committee of Key Stakeholders

A stroke committee—consisting of a telestroke coordinator, ED physicians and nurses, radiology staff, and EMS—proved essential to improving telestroke practices at GRU-14, GRU-17, MUSC-3, MUSC-7, and MUSC-8. Emphasizing the need for coordination, the chief of medical staff at MUSC-7 said, “We consider stroke to be a team event.” In some spokes, the committee also facilitated a cultural change. An ED physician at GRU-14 explained:

When I arrived here 3 years ago, we did not have a stroke care culture. The stroke committee took ownership of the stroke program and led the change in culture from within. Now, stroke is a source of identity for the hospital.

The committees met regularly to discuss issues and to find ways to enhance stroke readiness. The role of ED physicians and nurses in stroke committees was critical. In some spokes (eg, GRU-5), the nurses encouraged the ED physicians to initiate the REACH call, while at others (eg, GRU-14), the ED physicians themselves contacted the remote specialist. In all cases, however, the ED physicians made a decision (to treat locally or transfer patients) based on availability of local neurology support and neuro-ICU facilities in their hospital. Deliberate engagement of the local EMS in some spokes (MUSC-7 and GRU-14) improved stroke performance by reducing patient transportation time. Similarly, a pro-active EMS became an integral part of stroke care at MUSC-8. At MUSC-9, the hospital-owned EMS became the “voice of the hospital.”

#### Continuous Telestroke Process Improvement

Spokes with superior stroke performance (eg, GRU-14 and MUSC-7) focused on improving their stroke delivery processes. Their stroke committees had developed protocols and training procedures to sustain and improve urgent stroke care. Stroke care-related staff at GRU-14 and GRU-17 regularly exchanged best practices and updates with colleagues in other hospitals. The chief financial officer at GRU-17, who trained as a Six Sigma Master Black Belt, had initiated several quality improvement initiatives to improve stroke care. Over time, GRU-17 fostered shared responsibility for stroke care and created a systematic basis for continuous improvements. In contrast, GRU-8 did not have established routines or process improvement initiatives to develop their urgent stroke care capability. At GRU-14, MUSC-7, and MUSC-8, the process improvement initiatives helped achieve the coveted primary stroke center accreditation.

## Discussion

### Principal Results

The existing IT literature emphasizes how organizational factors enable technology utilization in key processes to enhance business performance [12-14]. Based on this general logic, our study highlights the organizational factors that drive telestroke assimilation at hub and spoke levels. Using data from 2 telestroke networks that operated in similar contexts and relied on the same technology, we investigated the variations in technology assimilation across spokes and zoomed in on organizational factors that could explain this variation.

**Figure 3:**
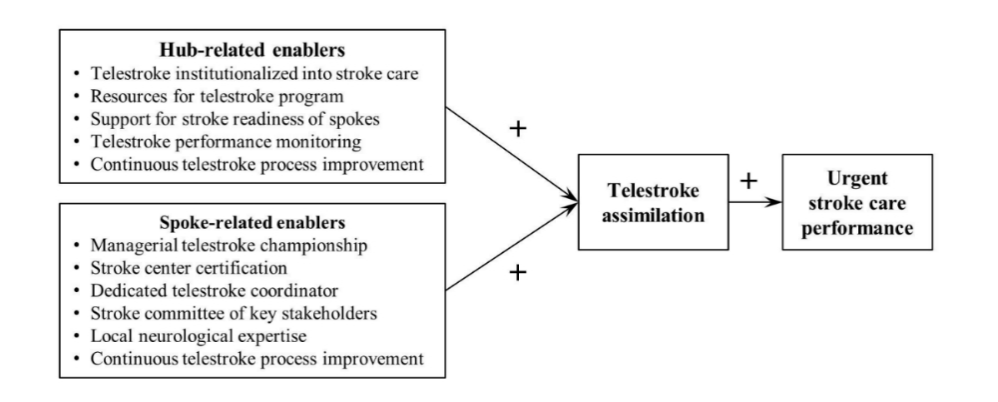
Proposed model of telestroke assimilation.

The identified hub factors included (1) institutionalization of telestroke by making the technology an integral part of stroke delivery, (2) providing required resources for telestroke program, (3) ongoing support for stroke readiness of spokes, (4) telestroke performance monitoring with site-specific feedback, and (5) continuous process improvement to improve telestroke delivery. Similarly, the identified spoke factors included (1) managerial telestroke championship, (2) stroke center certification, (3) dedicated telestroke coordinator, (4) stroke committee consisting of key stakeholders, (5) availability of local neurological expertise, and (6) continuous telestroke process improvement. These empirical findings suggest a telestroke assimilation model (Figure 3) in which specific hub and spoke factors enable increased use of telestroke technology for urgent stroke evaluation. Moreover, as several studies have established, improved urgent stroke evaluation and management—through tPA administration in ischemic strokes or neurosurgical interventions, as appropriate—greatly reduce the chance of severe disabilities [49]. Therefore, the proposed model includes urgent stroke care performance as the overall outcome.

### Comparison With Prior Work

Existing telestroke studies support some of our findings and related elements of the assimilation model. On the hub level, Cho et al [42] found the enabling effect of “institutionalization of telestroke into routine stroke delivery.” Similarly, considering “resources for telestroke,” Gogan and Garfield [50] found that effective deployment of organizational resources is critical to developing and improving telestroke services. However, few studies have so far examined hub-related organizational factors, such as telestroke process improvement, support for stroke readiness of spokes, and spoke-specific programs for performance monitoring.

On the spoke level, Rogove et al [30] found that lack of leadership support was a major barrier to telestroke, thus emphasizing the enabling role of “managerial telestroke championship.” O'Toole Jr. et al [29] identified the lack of “local neurological expertise” in rural areas as a major barrier to telestroke adoption and implementation. Other studies have pointed to the need for “continuous telestroke process improvement.” For example, Medeiros de Bustos et al [51] identified the lack of predefined procedures and uneven standards of evaluating stroke care quality as major challenges to telestroke utilization, and Gogan and Garfield [50] identified the need to create appropriate checklists and protocols for stroke care and to engage users in developing repeatable processes. Interestingly, although many studies point to the general need for internal and external coordination for stroke care [29,52], few studies have examined the role of a stroke committee of key stakeholders in directing such efforts or of a dedicated telestroke coordinator in facilitating day-to-day stroke delivery.

Thus, our findings add to the literature in a number of ways. To our knowledge, this is the first study to focus on telestroke assimilation as a key activity in determining how technology contributes to urgent stroke care performance. Second, we have distinguished between hub- and spoke-level factors as the key organizational antecedents to telestroke assimilation. This is particularly important because most studies have focused on spoke-related factors. Finally, we have leveraged our empirical findings to propose a comprehensive model of telestroke assimilation in hospitals that have already deployed the technology.

### Limitations

An important limitation relates to the scope of this study. We considered the impact of organizational factors on telestroke assimilation, but did not explore policy-related (eg, reimbursement and incentive structures), technology-related (eg, reliability, ease-of-use, broadband connectivity, and level of integration of telestroke with other IT in the hospital), or behavioral factors (such as physician attitudes toward thrombolysis and technology, and local neurologists’ buy-in). Furthermore, we assumed that the patient population characteristics were similar across the spoke hospitals’ service areas. It is also important to note that not all hospitals may have the financial resources to hire a neurologist or a dedicated stroke coordinator (which may explain their reluctance or inability to use telestroke). Our findings draw on a comparative case study of two telestroke networks involving a particular technology. Although a case study design has limited generalizability [33,34], it has the advantages of attention to organizational context, dynamics, and multiple stakeholder perspectives [53]. Accordingly, we have provided a rich description of the two networks to help researchers assess and transfer the findings to other settings [54]. We triangulated across data sources, checked against “hard facts” (eg, published documents), used multiple investigators, and iteratively sought feedback on our interpretations from key stakeholders [33,34]. This approach improves the study’s confirmability and credibility [54,55]. Finally, the *P* values reported in the results section need to be viewed in light of the low sample size, which affects statistical power and our ability to make meaningful inferences.

### Directions for Future Research

Our study suggests some future research directions. First, researchers can validate and improve the proposed telestroke assimilation model by considering additional factors (eg, policy-related, technological, and behavioral) across different networks. Second, researchers can adapt the model to examine postdeployment utilization of telemedicine and other IT (such as EMR and health information exchanges) in health care organizations. Third, the literature provides several examples of maturity models for IT adoption and assimilation. The term “maturity” relates to the degree of repeatability and optimization of processes, from ad hoc practices, to formally defined steps, to managed result metrics, to active optimization of processes [56]. Accordingly, researchers can leverage our findings to develop a stroke capability maturity model to assess a hospital’s current practices and to develop strategies to improve stroke care capability. Finally, researchers can identify and characterize the processes through which health care providers learn to co-create value through collaborative forms of IT.

### Conclusions

EDs in rural hospitals with limited neurological expertise face significant challenges in evaluating patients with stroke symptoms. These hospitals need to either transfer stroke patients to larger regional medical centers or hire local neurologists. Recent telemedicine innovations have enabled rural hospitals to connect virtually to regional medical centers for urgent stroke evaluation. However, many hospitals that have deployed telestroke have not assimilated the technology, that is, they have not integrated it into their regular stroke delivery processes. Consequently, neurologic expertise is not used optimally, opportunities for tPA administration may be lost, and patients are transferred out unnecessarily. Based on a detailed examination of variations in telestroke assimilation across two networks, this exploratory research proposes a telestroke assimilation model that includes specific hub- and spoke-related characteristics that can potentially increase IT assimilation by spokes and lead to improved stroke readiness.

## Abbreviations

CT: computed tomography
ED: emergency department
EMS: emergency medical services
GRU: Georgia Regents University
ICU: intensive care unit
IT: information technology
MUSC: Medical University of South Carolina
REACH: Remote Evaluation of Acute isCHemic stroke
tPA: tissue plasminogen activator
